# Chemical and Pharmacological Evaluation of Hulls of *Prunus dulcis* Nuts

**DOI:** 10.1155/2019/5861692

**Published:** 2019-11-22

**Authors:** Muhammad Nasimullah Qureshi, Sodik Numonov, Haji Akber Aisa

**Affiliations:** ^1^Department of Chemistry, University of Swabi, Anbar, Pakistan; ^2^Key Laboratory of Plant Resources and Chemistry in Arid Regions, Xinjiang Technical Institute of Physics and Chemistry, Chinese Academy of Sciences, Urumqi 830011, China; ^3^Research Institution “Chinese-Tajik Innovation Center for Natural Products”, Ayni St. 299/2, Dushanbe 734063, Tajikistan; ^4^Center for Research in Innovative Technologies, Academy of Sciences of the Republic of Tajikistan, Ayni St. 299/3, Dushanbe 734063, Tajikistan; ^5^Key Laboratory of Xinjiang Indigenous Medicinal Plants Resource Utilization, Xinjiang Technical Institute of Physics and Chemistry, Chinese Academy of Sciences, Urumqi 830011, China

## Abstract

Researchers have shown that the almond hulls, normally wasted after utilization of nuts, contain a number of biologically active compounds based on which the present study has been carried out. Focus is placed on the mass spectrometric determination of the analytes along with the estimation of total polyphenolic and total flavonoid contents in the 70% ethanol extract. After partitioning the 70% ethanol extract in hexane, chloroform, ethyl acetate, *n*-butanol, and water, all the extracts were evaluated for their antioxidant, antidiabetic, and antimicrobial activities. The results delivered total polyphenolic compounds as gallic acid equivalents (1% w/w) of the dried extract and total flavonoid contents as quercetin equivalents (0.2% w/w) of the dried extract. Mass spectrometric analysis resulted in the identification of 15 compounds containing various derivatives of (epi)catechin, chlorogenic acid, kaempferol, isorhamnetin and their glycosides, ursolic acid, amygdalactone, and benzoic acid derivatives. Antioxidant activity experiments showed that highest activity was found in *n*-butanol extract among the studied samples with IC_50_ value as 76.04 *μ*g/ml, while hexane and chloroform extracts were active against the PTP1B enzyme with IC_50_ values 9.66 *μ*g/ml and 37.95 *μ*g/ml, respectively. Hexane and chloroform fractions were active against *Staphylococcus aureus* with the zone of inhibition diameter 9 mm and 12 mm, respectively.

## 1. Introduction

Hulls and peels from the fruits and vegetables are being considered as a source for getting beneficial products such as phenolic compounds, which have proven antioxidant activity along with other pharmacological activities [1–4]. Almond nuts have been used since long for their various nutritional and pharmacological values as snacks or as a constituent for manufacturing processed food [2]. Almond hulls are removed for the utilization of nuts and are wasted without any use or used as feedstock in few cases. Researchers have shown that almond hulls are valuable byproducts of almond nuts, which contain a number of biologically active compounds such as triterpenoids, phenolic compounds, and their derivatives. Among the triterpenoids, betulinic acid, oleanolic acid, and ursolic acid have been identified, constituting about 1% of the hulls [5]. Flavan-3-ols, cinnamic acid, and hydroxybenzoic acid have been reported in almond hulls [1, 5, 6]. Glycosylated flavonols such as rhamnetin or isorhamnetin glycosides, quercetin glycosides, kaempferol glycosides [1, 7] and chlorogenic acid and their derivatives [5] have been identified in the extracts of almond hulls. Volatile constituents of the almond hulls have also been studied [8, 9].

The present study is conducted on the hulls of almonds collected from Kashgar area of China. Quantification of total polyphenolic compounds and total flavonoids were performed along with the identification of compounds through HPLC-MS/MS analysis in the 70% ethanol extract. Furthermore, all the prepared extracts were evaluated for their antioxidant and antimicrobial activities.

## 2. Materials and Methods

### 2.1. Chemicals and Reagents

Quercetin (98%), gallic acid (≥97%), aluminium chloride, sodium acetate, Folin-Ciocalteu reagent (2 N), DPPH, EDTA, and vitamin C were purchased from Sigma-Aldrich GmbH (Steinheim). Absolute ethanol, methanol, hexane, chloroform, ethyl acetate, and *n*-butanol were procured from Tianjin Baishi Chemical Company (Pvt), Urumqi, China. All the chemicals and reagents were of analytical grade, and double-distilled water was used throughout the experiments.

### 2.2. Collection of the Almond Fruit

Almond fruits (*Prunus dulcis*) were obtained from an almond farm in Kashghar, China. The fruits were authenticated, and the voucher sample has been deposited in the herbarium of the Xinjiang Technical Institute of Physics and Chemistry, Chinese Academy of Sciences, Urumqi 830011, P. R. China. Hulls were removed and ground to powder with particle size 50-300 mesh through a grinder (Model: 500A) from AO LI BANG, P. R. China.

### 2.3. Extraction

**Figure 1:**
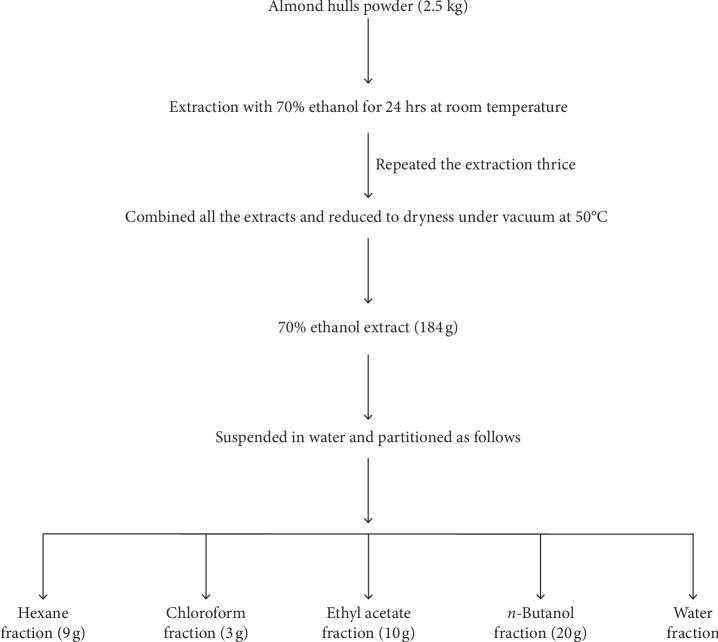
Flow sheet diagram of extraction and fractionation of almond hulls powder.

Almond hulls powder was extracted with 70% ethanol with the plant-to-solvent ratio of 1 : 10 for 24 hours at room temperature, and the extraction was repeated thrice. Combined extract was reduced to dryness under vacuum at 50°C. The dried 70% ethanol extract was suspended in 500 ml water and partitioned successively with hexane (3 × 500 ml), chloroform (3 × 500 ml), ethyl acetate (3 × 500 ml), and *n*-butanol (3 × 500 ml) producing hexane, chloroform, ethyl acetate, and *n*-butanol fractions. The remaining is the aqueous extract.  Figure 1 shows the flow sheet diagram of extraction and fractionation of almond hulls powder.

### 2.4. Determination of Total Polyphenolic Compounds and Total Flavonoids

One gram of the dried 70% ethanol extract of almond hulls was reconstituted in 20 ml of 70% methanol, and this extract was used for the determination of total polyphenolic compounds and total flavonoid contents. Folin-Ciocalteu method published in the literature [10, 11] was used for the estimation of total polyphenolic compounds as follows: FC reagent (2 N) was first diluted with water at a ratio of 1 : 10 and then 5 ml of the diluted FC reagent was thoroughly mixed with 1 ml of each of blank (water), extract, and the working standards solutions in test tubes separately. After 8 minutes of reaction, 4 ml of Na_2_CO_3_ solution (7.5%) was added to each mixture and mixed thoroughly. Samples were preserved for 2 hours at room temperature and kept away from strong light. Absorbance of these test solutions was read against the prepared blank at 740 nm by using a UV-visible spectrophotometer. Gallic acid was used as the reference standard, and working standards were prepared in the concentration range of 0.02 mg/ml to 0.2 mg/ml in water.

Total flavonoid contents were determined employing the method as stated in the published literature [11, 12]. 1 g of the dried extract was reconstituted in 20 ml of 70% ethanol and dried under vacuum. The obtained residue was dissolved in 10 ml of methanol, and this solution was used for further experiment. 1.5 ml of methanol was added to 0.5 ml of each of the plant extract, working standard solutions, and the blank (methanol) in test tubes. Flavonoid-aluminium complex was prepared in extract/standard test tubes by successive addition of 10% aluminium chloride (0.1 ml), 1 M potassium acetate (0.1 ml), and distilled water (2.8 ml). Solutions were thoroughly mixed at each step, and the absorbance of the reaction mixtures was measured after 30 minutes at 415 nm by using the UV-visible spectrophotometer. Working standard solutions of quercetin were prepared in the concentration range 0.01 mg/ml to 0.1 mg/ml in methanol.

### 2.5. HPLC-MS/MS Analysis

Analysis of 70% ethanol extract was performed on a linear ion-trap mass spectrometer (4000 Q TRAP) connected to the LC system through ESI interface from AB Sciex. Analyst 1.5 software was used to control LC-ESI-MS. The sample was chromatographed on a reversed-phase column (XBridge™ C18, particle size 5 *μ*m, 4.6 × 150 mm with guard column). Mobile phase consisted of A (1% formic acid in water) and B (1% formic acid in acetonitrile). Column temperature was kept as ambient, and a flow rate of 0.5 ml per minute was used. Gradient elution was started at 5% B and raised to 60% B in 30 minutes. The concentration of the mobile phase was changed to 100% B up to 35 minutes and hold at 100% B for 5 minutes. The whole analysis was completed in 40 minutes. MS was operated in negative ionization mode, and the scanning was performed in the *m*/*z* mass range values from 100 to 2000. Twenty microliters (20 *μ*l) of the sample was injected into the chromatographic column.

### 2.6. Antioxidant and Antidiabetic Activities

The antioxidant activity of all the extracts on DPPH (1,1-diphenyl-2-picrylhydrazyl) was determined according to the procedure published in the literature [13, 14]. The extracts were dissolved in DMSO in a concentration of 100 ppm. Sample solution (2.5 ml) was added to a 96-microwell plate. One milliliter of 0.3 mM DPPH solution in ethanol was added in each well to produce the test solutions. DMSO (1 ml) was used as a blank solution. A negative control sample was prepared by mixing 1 ml of DPPH solution with DMSO (2.5 ml). Ascorbic acid (vitamin C) was used as the positive control in this assay. All the solutions were kept in the dark at room temperature for 30 min. Absorbance was monitored at a wavelength of 517 nm, and the half maximal inhibitory concentration (IC_50_) was calculated using the observed absorbance values.

Antidiabetic activity of all the extracts was determined using the PTP1B enzyme inhibition assay according to the procedure published in the literature [15]. Test sample was prepared by dissolving 0.1 g of the dried extract in 1 ml of DMSO. 1 ml of the PTP1B protein solution (0.115 mg/mL) and 1 *μ*l of the test sample/positive control sample/DMSO were added to the 96-well plate containing 178 *μ*l of buffer solution [20 mM HEPES (4-(2-hydroxyethyl)-1-piperazine ethanesulfonic acid), 150 mM NaCl, and 1 mM EDTA (ethylenediaminetetraacetic acid)] and mixed them well. The 96-well plate was incubated at room temperature for 10 min, and then 20 *μ*l of pNPP solution (35 mM) was added. After 30 minutes of reaction in dark at 25°C, 10 *μ*l of 3 M NaOH solution was mixed. The absorbance was determined at 405 nm wavelength using SpectraMax MD5 (Molecular Devices, USA) and was corrected by measuring the increase in absorbance at 405 nm of the sample from the nonenzymatic hydrolysis of 35 mM pNPP obtained in the absence of PTP1B enzyme. Inhibition percentage (%) and IC_50_ value were calculated. PTP1B inhibitor was used as a control sample.

### 2.7. Antimicrobial Activity

Antimicrobial activities of the crude extracts were measured using the agar well diffusion method [16, 17]. Fungal and bacterial pathogens: *Candida albicans* (CA; ATCC10231), *E. coli* (EC; ATCC11229), and *Staphylococcus aureus* (SA; ATCC6538), were used as indicator strains for this analysis using ampicillin sodium salt and amphotericin B as standards [18]. These microorganisms were aseptically inoculated into appropriate liquid media and incubated at 37°C. After 16 h, the cells were centrifuged at 6000 rpm for 10 min and then suspended in sterile water. The different cells (1 ml) were added to appropriate agar media (100 ml) prior to plating, and the wells were made using an agar well borer. To these wells, extracts having 100 ppm concentrations were added and subsequently incubated at 37°C for 24 h. Zone of inhibitions were estimated by measuring the diameter of the microbial growth inhibition zone. Values were averaged from three independent experiments.

## 3. Results and Discussion

### 3.1. Total Polyphenolic Compounds and Total Flavonoid Contents

**Figure 2:**
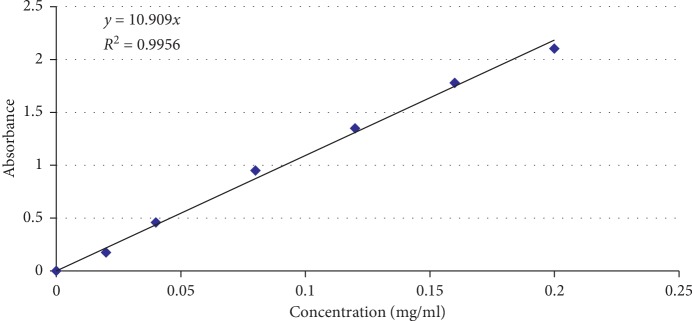
Calibration curve of gallic acid for the determination of total polyphenolic compounds.

Total polyphenolic compounds were calculated as gallic acid equivalent using the regression equation obtained from the calibration curve with an *R*^2^ value of 0.9956 (Figure 2). The values obtained from triplicate experiments were averaged resulting in the total amount of poyphenolic compounds as gallic acid equivalents as 1% w/w of the dried extract.

**Figure 3:**
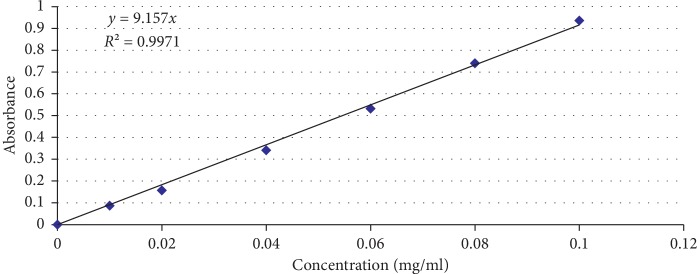
Calibration curve of quercetin for the determination of total flavonoid contents.

Total flavonoid contents were quantified through the procedure of reacting AlCl_3_ with the free flavonoids present in the extract, resulting in the Al-flavonoid complex formation with an absorption maximum at 415 nm. MS Excel sheet was used for constructing the calibration curve after observing the absorbance of the quercetin standard solutions in the range of 0.01 mg/mL to 0.1 mg/mL in methanol at 415 nm (Figure 3). Calibration curve with an *R*^2^ 0.9971 was obtained, and the experiment was repeated three times. This delivered an averaged value for the total flavonoid contents as quercetin equivalents as 0.2% w/w of the dried extract.

### 3.2. HPLC-MS/MS Analysis

**Figure 4:**
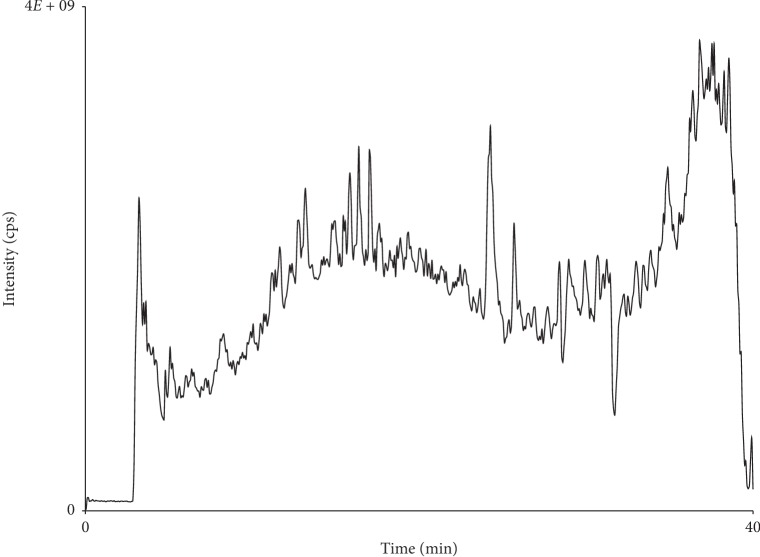
Total ion chromatogram of the 70% ethanolic extract of almond hulls.

Total ion chromatogram has been shown as Figure 4, while Table 1 shows the compounds identified in the 70% ethanol extract of almond hulls through LC-MS/MS analysis along with their peaks retention times, fragmentation pattern, and the references. The names of the compounds to the peaks were assigned based on matching the obtained fragmentation pattern of the analytes at the given retention time with those published in the literature as cited.

Protocatechuic acid gave a peak at 9.273 min with [M-H]^−^ ion at the *m*/*z* value 153 with an MS^2^ fragment at *m*/*z* 109 due to the loss of mass unit 44, which may be due to the removal of CO_2_ from pseudomolecular ion [M-H-CO_2_]^−^. Signals at 10.051, 10.687, and 13.658 min with [M-H]^−^ ion at the *m*/*z* value 577 gave fragmentation pattern similar to the (epi)catechin dimer as confirmed from the literature. The fragmentation pattern consisting of the main fragments at the *m*/*z* value 451 is due to heterocyclic ring fission [M-C_6_H_6_O_3_-H]^−^, 425 is due to retro-Diels–Alder cleavage [M-C_8_H_8_O_3_-H]^−^, 407 is due to subsequent dehydration [M-C_8_H_8_O_3_-H_2_O-H]^−^, and 289 which is due to [M_(epi)catechin_-H]^−^.

Trimeric (epi)catechin appeared at 10.616 with [M-H]^−^ ion at the *m*/*z* value 865. Main fragments in the fragmentation pattern are as follows: at *m*/*z* value 738 [M-C_6_H_6_O_3_-H]^−^, 713 [M-C_8_H_8_O_3_-H]^−^, and 695 [M-C_8_H_8_O_3_-H_2_O-H]^−^ and interflavanic bond breakage producing ions at *m*/*z* 577 and 289. Peaks at 11.099, 28.821, and 29.672 minutes were attributed to (epi)catechin with [M-H]^−^ ion at the *m*/*z* value 289. Chlorogenic acid with [M-H]^−^ ion at the *m*/*z* value 353 arose at 11.704 min, showing a fragment ion at *m*/*z* 335 due to the removal of water molecule. Peak at 22.167 min was assigned to 3-prenyl-4-O-*β*-glucopyranosyl oxy-4-hydroxy benzoic acid with [M-H]^−^ ion at the *m*/*z* value 367. Based on the [M-H]^−^ ions and their fragmentation pattern, peaks at 13.048 min and 14.893 min were assigned to kaempferol rhamnoside and kaempferol glucoside, respectively. Isorhamnetin rutinoside and isorhamnetin gave signals at 16.119 min and 16.187 min.

The peak at 20.207 min gave a fragmentation pattern that of singly hydrated chlorogenic acid. [M-H]^−^ ion at the *m*/*z* value 371 was due to hydrated molecule of chlorogenic acid, which lost a mass of 18 m.u. (water molecule) producing a signal for the *m*/*z* value 353, which is that of chlorogenic acid. Kaempferol showed a peak at 21.258 while (epi)catechin dihydrated molecules showed a peak at 23.085 and 26.093 min, showing signal at the *m*/*z* value 325, which on successive lose of 18 m.u., producing signals at 307 and 289. Mono hydrated (epi)catechin appeared at 26.02, 26.582, and 29.725 min. The peak at 31.371 min was assigned to ursolic acid with [M-H]^−^ ion at 455, while peaks at 37.803 and 39.084 min gave a fragmentation pattern similar to that of amygdalactone, producing [M-H]^−^ ion at *m*/*z* 295.

All the compounds shown in the table have already been identified in the almond hulls except dimeric, trimeric, and hydrated (epi)catechin derivatives. Among the three triterpenoids such as betulinic acid, oleanolic acid, and ursolic acid which have been identified previously in the almond hulls, we could only identify ursolic acid in our study. Ursolic acid has shown various pharmacological activities such as antitumor, anti-inflammatory, antihyperlipidemic, anti-HIV, and hepatoprotective [2, 6].

### 3.3. Pharmacological Activities

#### 3.3.1. Antioxidant Activity

DPPH is a stable radical and mostly employed for the evaluation of antioxidant activity. It has a characteristic absorption at the wavelength 517 nm, which decreases when exposed to some radical scavengers. Lower absorption at the wavelength 517 nm delivers lower IC_50_ value indicating stronger activity. Antioxidant activities of the seven samples/extracts were tested using the DPPH assay procedure. All the samples showed very low antiradical activity as compared to the standard used as vitamin C with an IC_50_ value 5.34 *μ*g/ml (Table 2). Among the samples, *n*-butanol fraction showed highest activity with IC_50_ value 76.04 *μ*g/ml. IC_50_ values of chloroform, ethyl acetate, and 70% ethanol extracts were 128.17, 148.32, and 167.11 *μ*g/ml, respectively. IC_50_ values of the hexane and water fractions were beyond 500 *μ*g/ml.

#### 3.3.2. Antidiabetic Activity

Antidiabetic activity was determined using the protein tyrosine phosphatase-1B (PTP1B) inhibition procedure. Hexane and chloroform fractions showed antidiabetic (PTP1B inhibition) activity with an IC_50_ 9.66 *μ*g/ml and 37.95 *μ*g/ml, respectively (Table 2). PTP1B inhibitor showed inhibition with an IC_50_ 1.46 *μ*g/ml. Remaining extracts were inactive.

#### 3.3.3. Antibacterial Activities

All the seven samples were screened against the three microbial strains: *Candida albicans* (CA; ATCC10231; fungus), *Escherichia coli* (EC; ATCC11229; gram-negative bacteria), and *Staphylococcus aureus* (SA; ATCC6538; gram-positive bacteria) using ampicillin sodium salt and amphotericin B as standards. No any extract showed activity against the CA and EC, while hexane and chloroform fractions delivered activity against the SA with the zone of inhibition diameters 9 mm and 12 mm, respectively, in comparison with the standard ampicillin sodium salt, which yielded 19 mm inhibition zone diameter.

## 4. Conclusion

These analyses confirmed that the almond waste, i.e., hulls, is a rich source of pharmacological active metabolites, based on which the hulls can be used in various pharmaceutical preparations. Furthermore, the use of hulls for obtaining these chemicals can also reduce the cost incurred on the wastage of these hulls for those industries producing almond-based products.

## Figures and Tables

**Table 1:** Results of the LC-MS/MS analysis of the 70% ethanol extract of almond hulls.

No	RT (min)	Fragmentation pattern	Name	Reference
1	9.273	152.9, 109	Protocatechuic acid (dihydroxybenzoic acid)	[6]
2	10.051, 10.687, 13.658	577.2, 451, 439, 425, 407, 289.2	(epi)Catechin-(epi)catechin	[15]
3	10.616	865.3, 739, 713, 695, 577, 451.2, 425, 407, 289.1	(epi)Catechin-(epi)catechin-(epi)catechin	[15]
4	11.099, 28.821, 29.672	289.1	Catechin	[6]
5	11.704	353.1, 335.3	Chlorogenic acid	[1]
6	13.048	431.3, 285.2	Kaempferol rhamnoside	[7]
7	16.119	623.4, 315.1, 299	Isorhamnetin rutinoside	[15]
8	16.187	315.2, 297.4	Isorhamnetin	[15]
9	20.207	371.2, 353.2	Chlorogenic acid monohydrated	[19]
10	21.258	284.9	Kaempferol	[7]
11	22.167	367.1, 352, 336.2, 321.1, 306.2, 277.8	3-Prenyl-4-O-*β*-glucopyranosyl oxy-4-hydroxy benzoic acid	[6]
12	23.085, 26.093	325.2, 307, 289.2	(epi)Catechin dihydrated	[20]
13	26.02, 26.582, 29.725	307.3, 289.1	(epi)Catechin monohydrated	[20]
14	31.371	455.2	Ursolic acid	[6]
15	37.803, 39.084	295.2, 277.1	Amygdalactone	[21]

**Table 2:** Antioxidant and antidiabetic activities of *Prunus dulcis* hull extracts.

Sample	Antioxidant activity IC_50_ (*μ*g/ml)	Antidiabetic activity IC_50_ (*μ*g/ml)
70% ethanol extract	167.11	Inactive
Hexane fraction	>500	9.66 ± 0.42
Chloroform fraction	128.17	37.95 ± 0.14
Ethyl acetate fraction	148.32	Inactive
*n*-Butanol fraction	76.04	Inactive
Water fraction	>500	Inactive
Vitamin C	5.34 ± 0.42 (*μ*g/ml)	—
PTP1B inhibitor	—	1.46 ± 0.40

## Data Availability

The chemical analysis and pharmacological evaluation data of the shells of *Prunus dulcis* nuts used to support the findings of this study are included within the article.
